# AI Use in Mammography for Diagnosing Metachronous Contralateral Breast Cancer

**DOI:** 10.3390/jimaging10090211

**Published:** 2024-08-28

**Authors:** Mio Adachi, Tomoyuki Fujioka, Toshiyuki Ishiba, Miyako Nara, Sakiko Maruya, Kumiko Hayashi, Yuichi Kumaki, Emi Yamaga, Leona Katsuta, Du Hao, Mikael Hartman, Feng Mengling, Goshi Oda, Kazunori Kubota, Ukihide Tateishi

**Affiliations:** 1 Department of Breast Surgery, Tokyo Medical and Dental University Hospital, Tokyo 113-8510, Japan; adcsrg2@tmd.ac.jp (M.A.); ishiba.srg2@tmd.ac.jp (T.I.); maruya.sakiko@tmd.ac.jp (S.M.); hayashi.srg2@tmd.ac.jp (K.H.); kumaki.srg2@tmd.ac.jp (Y.K.); odasrg2@tmd.ac.jp (G.O.); 2 Department of Diagnostic Radiology, Tokyo Medical and Dental University Hospital, Tokyo 113-8510, Japan; ymgdrnm@tmd.ac.jp (E.Y.); leonah@jcom.home.ne.jp (L.K.); ttisdrnm@tmd.ac.jp (U.T.); 3 Ohtsuka Breast Care Clinic, Tokyo 121-0813, Japan; miyako641@gmail.com; 4 Saw Swee Hock School of Public Health, National University of Singapore, National University Health System, Singapore 119074, Singapore; duhao@fathomx.co (D.H.); mikael_hartman@nuhs.edu.sg (M.H.); ephfm@nus.edu.sg (F.M.); 5 Department of Surgery, National University Hospital, National University Health System, Singapore 119074, Singapore; 6 Institute of Data Science, National University of Singapore, Singapore 117597, Singapore; 7 Department of Radiology, Dokkyo Medical University Saitama Medical Center, Saitama 343-8555, Japan; kubotard@dokkyomed.ac.jp

**Keywords:** breast cancer, mammography, artificial intelligence, metachronous contralateral breast cancer

## Abstract

Although several studies have been conducted on artificial intelligence (AI) use in mammography (MG), there is still a paucity of research on the diagnosis of metachronous bilateral breast cancer (BC), which is typically more challenging to diagnose. This study aimed to determine whether AI could enhance BC detection, achieving earlier or more accurate diagnoses than radiologists in cases of metachronous contralateral BC. We included patients who underwent unilateral BC surgery and subsequently developed contralateral BC. This retrospective study evaluated the AI-supported MG diagnostic system called FxMammo™. We evaluated the capability of FxMammo™ (FathomX Pte Ltd., Singapore) to diagnose BC more accurately or earlier than radiologists’ assessments. This evaluation was supplemented by reviewing MG readings made by radiologists. Out of 1101 patients who underwent surgery, 10 who had initially undergone a partial mastectomy and later developed contralateral BC were analyzed. The AI system identified malignancies in six cases (60%), while radiologists identified five cases (50%). Notably, two cases (20%) were diagnosed solely by the AI system. Additionally, for these cases, the AI system had identified malignancies a year before the conventional diagnosis. This study highlights the AI system’s effectiveness in diagnosing metachronous contralateral BC via MG. In some cases, the AI system consistently diagnosed cancer earlier than radiological assessments.

## 1. Introduction

In 2020, an estimated 2.2 million people were diagnosed with breast cancer (BC), making it the most common malignant condition among women in Japan [1]. BC accounted for over 68,000 deaths that year, ranking it as the fifth leading cause of cancer-related deaths worldwide [1].

Mammography (MG) is widely utilized for BC screening, and its use has been associated with reduced mortality rates [2]. However, MG presents several challenges. Dense breast tissue, which is prevalent among young Asian women, can obscure cancer detection on MG and diminish its sensitivity [3]. Additionally, the risk of developing primary contralateral BC is estimated to be two to six times higher than that of developing a second BC in the general population [4,5,6]. Women under 50 years of age who develop contralateral BC within five years of their first diagnosis face a mortality risk that is 3.9 times greater than that faced by those with unilateral cancer [7]. Although the combined use of MG and Magnetic Resonance Imaging (MRI) shows 67% sensitivity and 50% specificity for detecting metachronous contralateral BC post-unilateral surgery [8], the guidelines currently recommend only physical exams and MG every 6–12 months for postoperative surveillance without endorsing further imaging tests [9,10].

In Japan, MRI surveillance after unilateral BC surgery is generally not covered by insurance, except in cases such as hereditary breast and ovarian cancer syndrome (HBOC) [9,10]. Recent advancements in deep learning-based artificial intelligence (AI) have significantly outpaced traditional computer-aided detection systems in breast imaging diagnostics [11]. Studies have shown that AI can facilitate the work of radiologists without compromising diagnostic quality [12]. Despite most people undergoing screening turning out to be cancer-free, AI systems are capable of identifying normal mammary glands and can be used for either primary or secondary readings, potentially enhancing screening efficiency [13,14,15,16]. AI also holds promise for pre-diagnostically identifying high-risk BC cases, potentially reducing intermediate-stage diagnosis and false-negative rates [16,17,18]. After unilateral BC surgery, MG interpretation becomes more complex. Usually, when reading MG, we compare the left and right sides. However, comparison is difficult in the metachronous bilateral BC setting, and there is no method of MG reading that is limited to unilateral MG. Variations in the mammary gland background and the necessity of comparing ipsilateral images to contralateral ones complicate image assessments. Postoperative deformities and calcifications can preclude the use of the operated breast for comparative analyses, thereby increasing the diagnostic burden on radiologists and heightening patient anxiety about developing contralateral BC. Bilateral BC may also indicate HBOC [19].

Despite its limitations, routine MG is currently the only surveillance method that guarantees the early detection of contralateral BC. Given these challenges, this study explores the potential of the AI system known as FxMammo™ (FathomX, Singapore), a mammography diagnostic support AI, to surpass radiologists in the accurate and early detection of metachronous contralateral BC using MG. This study aimed to explore the possibility of using the AI system for the accurate and early detection of metachronous contralateral BC.

## 2. Materials and Methods

### 2.1. Patients

**Figure 1:**
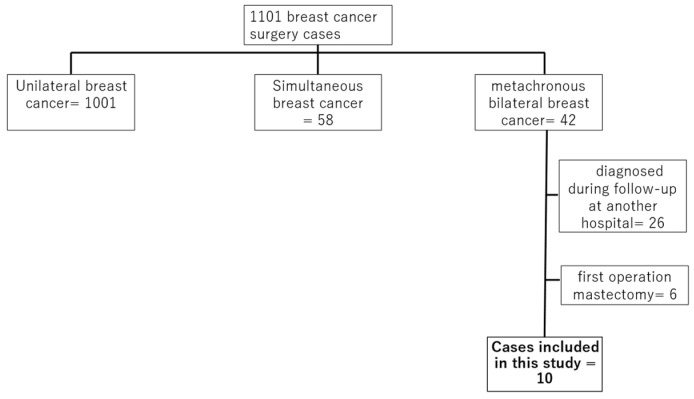
Algorithm for the cases included in this study. The number of cases operated on at the hospital was 1101. There were 1101 cases of unilateral breast cancer, 58 cases of simultaneous bilateral breast cancer, and 42 cases of heterochronic bilateral breast cancer. Twenty-six cases of contralateral breast cancer were noted during follow-up at other hospitals, and six cases of total resection were performed as the initial surgical procedure, making ten cases eligible for this study.

This is a retrospective study. A total of 1101 patients underwent BC surgery at Tokyo Medical and Dental University Hospital from January 2014 to December 2022. Among these, 42 cases of metachronous bilateral BC were identified. Twenty-six patients were diagnosed with contralateral BC at other institutions during follow-up, while sixteen were diagnosed and underwent contralateral BC surgery after the initial surgical procedure conducted at our hospital. Cases in which total mastectomy was performed as the initial surgical procedure were excluded due to the inability of the AI system to align left and right images. Therefore, six patients were excluded, leaving ten cases eligible for inclusion in this retrospective study (Figure 1).

### 2.2. Ethical Approval and Consent to Participate

This study was conducted per the principles outlined in the Declaration of Helsinki, the Clinical Research Act (Act No. 16 of 2017), the Enforcement Regulations of the Clinical Research Act (Ministry of Health, Labor and Welfare Ordinance No. 17 of 2018), and relevant notices. Ethical approval was obtained from the Ethics Review Committee of our hospital (approval ID: M2019-232, approval date: 13 December 2019). Informed consent was obtained from all patients regarding the use of their clinical data for research purposes.

### 2.3. Data Collection

Clinical information and pathological data were collected from medical records retrospectively. Image studies and diagnostic reports were obtained from the radiology reporting system. The imaging modalities utilized included MG, ultrasound (US), and MRI conducted before surgery for contralateral BC. MG used 2D diagnosis without tomosynthesis. The imaging diagnosis was evaluated by Japanese radiologists according to breast imaging reporting and data system (BI-RADS) categories [19]. The BI-RADS categories were collected from the reporting system as diagnosed by the radiologists at the time of imaging. Malignancy was defined as BI-RADS category 4 or higher. Pathological diagnoses were made by physicians specialized in Japanese pathology.

### 2.4. The AI System

**Figure 2:**
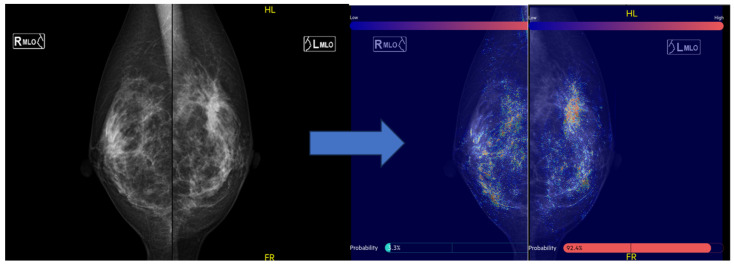
Displaying images in FxMammo. An image of the mediolateral oblique of mammography is shown on the left. A spiculated mass is seen in the left upper area. On the right is the result of the AI system analysis, with the areas of interest to the AI system indicated by the colors in the heatmap. The malignancy percentage is shown on the left and right sides, respectively (**right**: 3.3%; **left**: 94.2%).

We used the MG AI system FxMammo® (FathomX Pte Ltd., Singapore). The AI system is based on deep learning and has been put into practical use in Singapore and other countries. The mechanism of FxMammo has been described in previous studies. The AI system is based on the VGG-16 network [20]. The VGG network is one of the most used feature extractors in medical imaging classification [21]. The AI was created by collecting 17,769 cases (of which 45% were malignant) from 10 institutions in Taiwan, Thailand, Singapore, Hong Kong, China, Malaysia, and Japan. Since 2019, we have been collaborating with the National University of Singapore to develop MG AI for Asian women. This AI model, which utilizes CNNs and graph convolutional networks, quantifies the probability of malignancy and highlights areas of interest on a heat map when mammography images are uploaded. The model demonstrated a high Area Under the Curve (AUC) of 0.902 for BC detection [22]. If we assume a cancer miss rate of 3%, it is estimated that up to 38% of normal MG could be safely excluded from human review. In Japan, the AI system has not been approved for clinical use and it is used for research purposes. Four MG images (craniocaudal [CC], mediolateral oblique [MLO], left, and right) taken before surgery for heterochronic contralateral BC were transferred from the reporting system to the AI system where the MG data were analyzed. The threshold value was set to 40.0%, (91.5% sensitivity and 82.0% specificity). The AI system indicated the probability of malignancy for each of the four cards as a percentage. In addition, areas in which the AI system was interested were displayed in color on a heat map. The areas on which the AI system focused when analyzing the image are visually shown as a heat map (Figure 2).

### 2.5. Postoperative Surveillance

Postoperative BC surveillance at our hospital basically includes annual MG and US. MRI is performed when BC is strongly suspected by MG or US or after it is diagnosed via biopsy with histopathology. However, the interval of surveillance may become wider or narrower depending on the patients’ reasons. MG (CC and MLO) was performed bilaterally. US was performed by radiologists specialized in breast imaging diagnosis. A bilateral mammary MRI was acquired using a 3.0-T system with a breast coil and with the patient in the prone position. The unenhanced and enhanced phases were acquired at 1, 2, and 6 min in the axial plane after an intravenous bolus injection of gadolinium (0.1 mL/kg), using a fat-suppressed T1-weighted sequence (TR/TE = 6.5/2.4, flip angle = 10°, 2 mm thick section, 512 × 512 matrix, 360 mm field of view). The number of years until contralateral surgery was recorded as a whole number.

### 2.6. Diagnostic Imaging and Comparison with the AI System

For each image, the possibility of malignancy was diagnosed per BI-RADS. In cases where the AI system diagnosis differed from the interpretation of radiologists, the images and pathology were compared and examined in detail.

For each image, the possibility of malignancy was determined per BI-RADS. In cases where the AI system diagnosis differed from the radiologists’ interpretation, the images and pathology were compared and examined in detail. Based on the results, the BC detection rate for each modality was calculated. All the analyses were conducted using the EZR software package version 1.31 (Saitama Medical Center, Jichi Medical University, Saitama, Japan) [23].

Postoperative BC surveillance at our hospital, which is performed once a year, includes MG and US. MRI is performed when BC is strongly suspected by MG or US or after BC is diagnosed via biopsy with histopathology. However, the interval of surveillance may become wider or narrower depending on the patient’s reasons. MG (CC and MLO) was performed bilaterally. US was performed by radiologists specialized in breast imaging diagnosis. A bilateral mammary MRI was acquired using a 3.0-T system with a breast coil and the patient in the prone position. The unenhanced and enhanced phases were acquired at 1, 2, and 6 min in the axial plane after intravenous bolus injections of gadolinium (0.1 mL/kg), using a fat-suppressed T1-weighted sequence (TR/TE = 6.5/2.4, flip angle = 10°, 2 mm thick section, 512 × 512 matrix, 360 mm field of view). The number of years until contralateral surgery was recorded as a whole number.

### 2.7. Statical Analysis

Based on the analysis results, the BC detection rate for each modality was calculated. All statistical analyses were conducted using the EZR version 1.31 (Saitama Medical Center, Jichi Medical University, Saitama, Japan) [23].

## 3. Results

### 3.1. Patient Characteristics

In this study, we included the cases of ten patients, all of whom were Japanese females. Table 1 presents the clinical and pathological features of the disease at the time of the first surgery. The median age of our participants at the first diagnosis of BC was 68 years (range: 40–74 years). There were 3 cases (30%) at Stage 0, 4 cases (40%) at Stage I, and 3 cases (30%) at Stage II. Chemotherapy was administered to 4 cases (40%), and endocrine therapy was administered to 6 cases (60%). The preserved breasts were irradiated in all cases.

Table 2 presents the clinical and pathological features of contralateral breast cancer. The median time required for contralateral breast cancer to develop was 8 years (range: 2–10 years). The T classification ranged from Tis in 2 cases (20%), T1 in 7 cases (70%), T2 in 1 case (10%), Stage 0 in 2 cases (20%), Stage I in 7 cases (70%), and Stage IIA in 1 case (10%). The histological types were invasive ductal carcinoma (IDC) in 6 cases (60%), ductal carcinoma in situ (DCIS) in 2 cases (20%), apocrine carcinoma in 1 case (10%), and invasive lobular carcinoma (ILC) in 1 case (10%). Biology was luminal in 4 cases (40%), human epidermal growth factor receptor (HER) 2 in 1 case (10%), luminal-HER2 in 1 case (10%), and triple-negative breast cancer (TNBC) in 4 cases (40%).

### 3.2. Imaging Findings at the Time of Diagnosis

**Figure 3:**
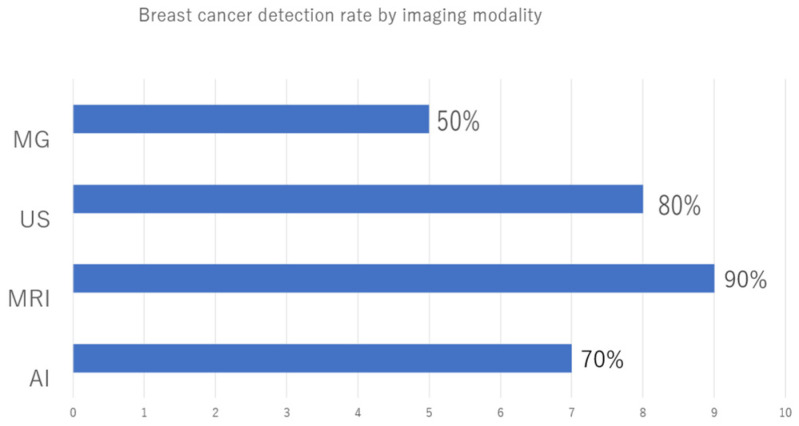
Breast cancer detection rate by imaging modality. Mammography (MG), ultrasonography (US), Magnetic Resonance Imaging (MRI), and the artificial intelligence (AI) system to diagnose the degree of malignancy. The highest diagnostic accuracy was 90% for MRI, followed by US, AI systems, and MG read by radiologists, in that order.

Table 3 presents the imaging findings at the time of diagnosis of contralateral BC. The MG categories according to BI-RADS were 1 in 4 cases (40%), 2 in 1 case (10%), 4 in 3 cases (30%), and 5 in 2 cases (20%). The US categories according to BI-RADS were 1 in 2 cases (20%), 4 in 5 cases (50%), and 5 in 3 cases (30%). The MRI categories according to BI-RADS were 1 in 1 case (10%) and 4 in 9 cases (90%). In cases 1 to 8, the lesions could be identified by US; therefore, this imaging modality was used to perform preoperative histological diagnoses. In case 9, the lesion could be identified only by MRI, so an MRI-guided biopsy was performed. In case 10, the preoperative diagnosis was Paget’s disease based on abrasive cytology. Although no lesions were found via breast imaging, postoperative specimens determined that there was DCIS within the breast. Accuracy was calculated for each imaging modality and the AI system. The accuracy for each imaging modality and the AI system was calculated, with MG at 50%, the AI system at 70%, US at 80%, and MRI at 90%, with MRI having the highest accuracy, followed by US, the AI system, and radiologist readings of MG (Figure 3).

### 3.3. Diagnosis by the AI System and Comparison with Past Images

Table 4 shows the results of the analysis of the diagnosis of MG heterochronic bilateral BC using the AI system. In cases 1–6, the diagnosis by the AI system indicated a possibility of malignancy. In cases 1 and 2, where the readings of radiologists indicated no possibility of malignancy, only the AI system diagnosed possible malignancy. We reviewed the MG images of these two cases. The area of interest of the AI system was identified as the focal asymmetric density (FAD) with increased density compared with other areas upon viewing after the final diagnosis. In cases 3, 4, 5, and 6, the AI and radiologists both diagnosed malignancy. Two cases involved masses and two others involved calcifications (Table 3). In case 7, only the radiologists diagnosed a mass visible only in the MLO direction as potentially malignant, which was a low-density mass. In cases 8, 9, and 10, the radiologists and the AI system both found no signs of malignancy. For cases 1–6, one MG image prior to the time of diagnosis was analyzed to verify whether the AI system could diagnose BC earlier (Table 5). In most cases, there was no difference between the diagnosis of the AI system and that of the radiologists. However, in cases 1 and 2, the AI system had previously diagnosed malignancy.

### 3.4. Representative Case

**Figure 4:**
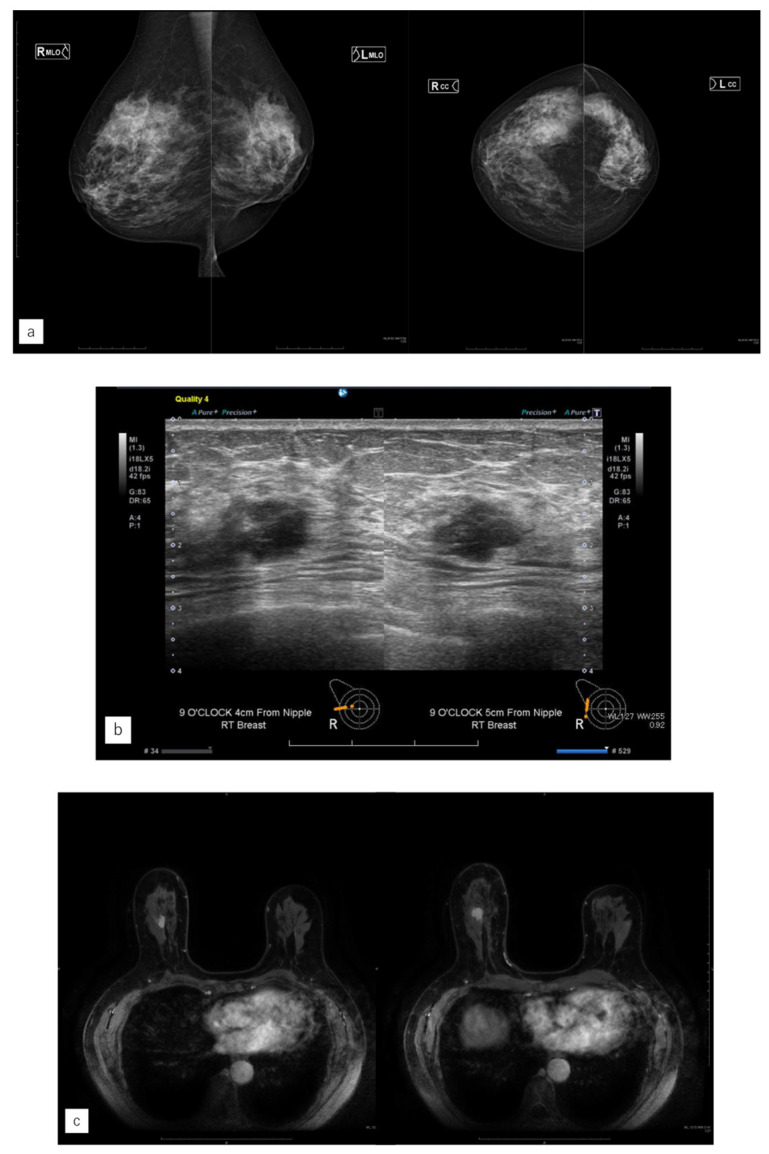

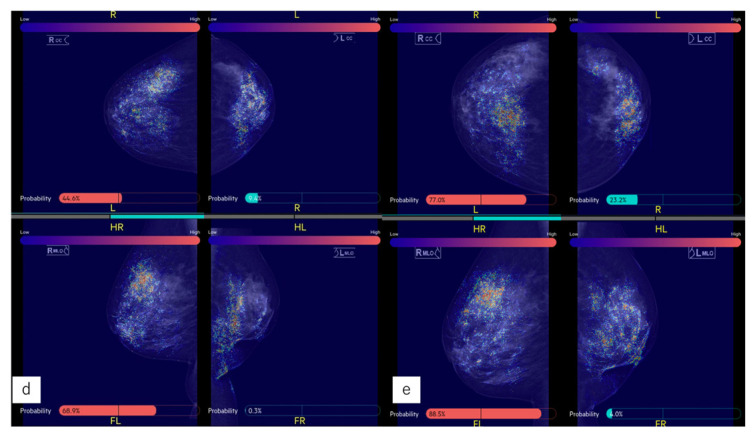
Representative case (case 1). (**a**) MG. (**b**) US. (**c**) MRI. (**d**) AI diagnosis. (**e**) Previous AI diagnosis. A 55-year-old woman had left-sided breast cancer. Eight years later, she was diagnosed with right-sided breast cancer. It was Lumina human epidermal growth factor receptor 2 with 15 mm of invasive cancer and 15 mm of non-invasive cancer. (**a**) There were no malignant findings on the right side of mammography (MG). (**b**) Ultrasonography revealed a hypoechoic mass in the right outer area. (**c**) Magnetic resonance imaging revealed a contrast-enhanced mass measuring 37 mm in the right outer area. (**d**) The artificial intelligence (AI) system diagnosed malignancy in the right breast based on MG at the time of diagnosis. The malignancy percentage is shown on the left and right sides (CC: right 44.6, left 9.4%, MLO: right 68.9%, left 0.3%). (**e**) The AI system also showed areas of interest in MG before the diagnosis, and it was diagnosed as possibly malignant. The malignancy percentage of the right side is CC in 77.0% and MLO in 88.5%.

**Figure 5:**
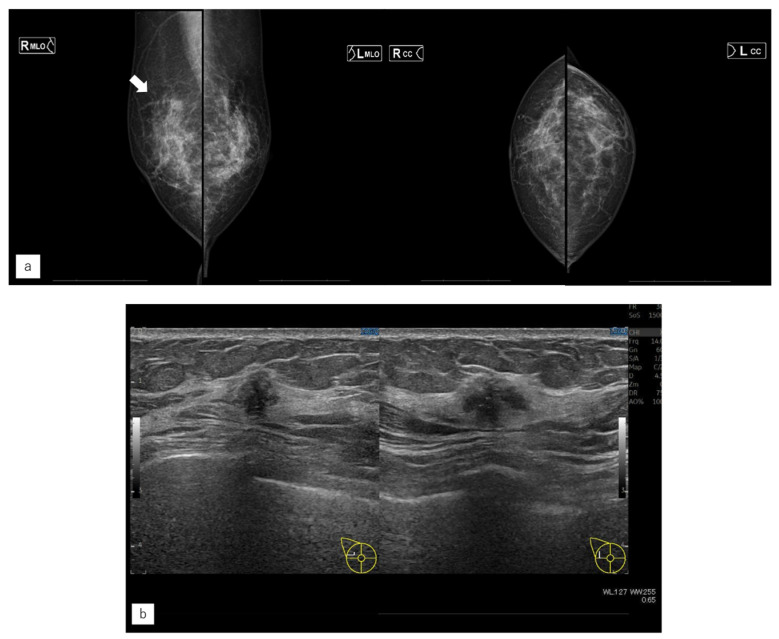

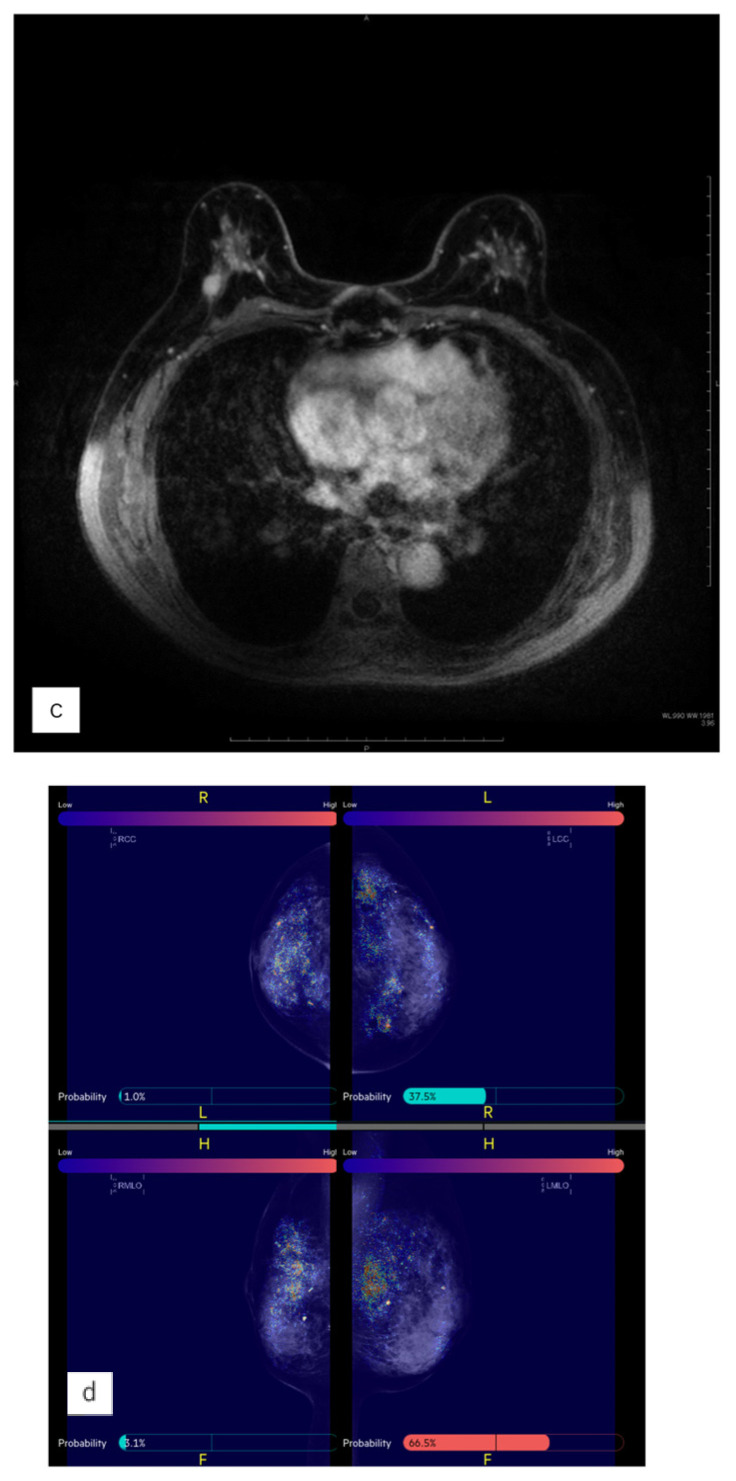
Representative case (case 7). (**a**) MG. (**b**) US. (**c**) MRI. (**d**) AI diagnosis. A 63-year-old woman had left-sided breast cancer. Two years later, she was diagnosed with right breast cancer. It was T1c, triple-negative breast cancer. (**a**) A mass was found in the right upper quadrant via mammography (MG) and diagnosed as a breast imaging reporting and data system (BI-RADS) Category 4. (**b**) Ultrasonography revealed a hypoechoic mass in the right upper outer quadrant. (**c**) Magnetic Resonance Imaging revealed a contrast-enhanced mass in the left upper outer quadrant. (**d**) The artificial intelligence system detected no malignancy. The mass visible on MG was not seen on MG a year earlier. Although the mass was of the same density as the background mammary gland, the radiologists found it to be possibly malignant upon comparison and reading. The malignancy percentage is shown on the left and right sides, respectively (CC: right 1.0%, left 37.5%, MLO: right 3.1%, left 66.5%).

Case 1, which only the AI system could diagnose, was examined in detail. In case 1, the AI system diagnosed a malignant finding in the right C area, considering it as the region of interest (Figure 4). In case 7, only the radiologists diagnosed a potentially malignant mass using MG (Figure 5). Compared with the previous year, it appeared as FAD. Radiologists diagnosed it as BI-RADS category 4, while AI detected no malignant findings. 

## 4. Discussion

In this study, we investigated the efficacy of an AI system for the MG diagnosis of asynchronous contralateral BC. By comparing the diagnostic abilities of radiologists with that of the AI system, we found that in two out of ten cases (20%), only the AI system identified potential malignancy, which had also been similarly assessed by the AI prior to mammographic diagnosis. Additionally, the radiologists and the AI system concurred in the absence of malignancy signs in three cases (30%), which were diagnosed complementarily with US, MRI, and physical examinations. The primary reason for concordant diagnoses between radiologists and the AI system seems to be that the AI training data were created by humans, suggesting that the AI system may mimic human diagnostic patterns.

The significance of this study can be divided into three major aspects. First, the use of an AI system could enhance diagnostic accuracy and facilitate the early detection of BC. Per NCCN guidelines [9], annual MG is recommended for surveillance in postoperative BC patients who face a two- to six-fold higher risk of contralateral BC compared with non-cancer patients [4,5,6]. Particularly in cases of contralateral surgeries, the lack of comparative images complicates diagnosis; hence, the AI system could effectively supplement this limitation. Some instances where the AI system detected malignancy solely on past MG highlight its potential for use as a diagnostic aid.

Second, the implementation of an AI system could reduce the workload of radiologists and relieve their stress. By undertaking efficient image analyses, AI allows radiologists to focus on more critical cases, potentially improving diagnostic accuracy. Additionally, referencing AI analyses could boost the confidence of radiologists in their diagnoses, thereby mitigating their psychological stress. With advancements in technology, the precision of AI diagnostics may evolve, possibly transforming the role of radiologists into a more efficient diagnostic process.

Third, the AI system could offer psychological reassurance to patients. Patients with a history of BC often fear recurrence or de novo cancer development; therefore, detailed image analysis by AI could help them better understand their health status and gain reassurance [24]. Particularly, AI-generated heatmaps could visually demonstrate suspicious areas, potentially alleviating patient anxiety.

In recent years, AI technology has advanced and is being applied to various medical imaging modalities [25,26,27,28,29,30]. In BC imaging diagnosis, its usefulness has been demonstrated in multiple modalities, including MG, US, MRI, and positron emission tomography [31,32,33,34,35].

To date, only one study has reported the use of AI for postoperative BC surveillance. Out of 314 cases, three were cases of heterochronic contralateral BC. The recall rate for contralateral BC was significantly lower with the combination of mammography and an AI system (1.5%) compared with mammography alone (6.6%, *p* < 0.01). The accuracy was significantly higher with the combination of mammography and an AI system (97.1%) compared with mammography alone (92.5%, *p* < 0.001) One-tailed *p*-values had a statistical significance threshold of 0.05. All analyses were performed using R version 3.5.1 [36]. AI use resulted in a decreased recall rate and improved accuracy, which mirrors the trend observed in this study. We only considered cases of heterochronic contralateral BC; however, a larger-scale validation including non-recurrent cases is necessary to further verify the AI system’s utility.

This time, we did not measure the time it took for radiologists and the AI system to make a diagnosis. As a future research topic, we would like to measure the time it takes to make a diagnosis. We also would like to conduct prospective studies and increase the number of cases.

Nevertheless, this study had several limitations. First, it is a retrospective, single-center study with a small sample size. Additionally, while the AI system is approved for MG screening in other countries, it is still not approved for clinical use in Japan as it is only available for research there. There is little evidence of its use in postoperative patients. Future studies should collect more mammographic data from multiple facilities to conduct prospective validations of the AI system. This study’s small sample size also limited its ability to account for diversity in patient demographics such as age and ethnicity and genetic factors, which may have affected the AI system’s performance. In this study, FxMammo™, the AI system itself, may have a bias in that it only targets Asian women. This bias should be addressed in future studies.

## 5. Conclusions

In the mammographic diagnosis of asynchronous contralateral BC, the AI system demonstrated the ability to identify signs of malignancy that radiologists may overlook. These results suggest that the AI system could contribute to the early detection and enhanced accuracy of BC diagnosis.

## Figures and Tables

**Table 1:** Clinical and pathological characteristics of our participants at the time of the first surgery.

No of Case	Age	TNM	Stage	Procedure	Axillary Lymph Node	Histology	Biology	Chemotherapy	Endocrine Therapy	RT
1	55	T2N1M0	0	Bp	Ax	IDC	HER2	○	×	○
2	71	T1micN0M0	I	Bp	None	A pocrine	HER2	×	×	○
3	68	T1cN0M0	I	Bp	SNB	IDC	Luminal	×	○	○
4	70	T1bN1M0	I	Bp	None	IDC	Luminal	×	○	○
5	68	T2M0M0	IIA	Bp	Ax	IDC	Luminal	○	○	○
6	60	T1cN0M0	I	Bp	SNB	IDC	Luminal	○	○	○
7	63	TisN0M0	0	Bp	SNB	DCIS	Luminal	×	×	○
8	40	TisN0M0	0	Bp	SNB	DCIS	Luminal	×	×	○
9	66	T1cN0M0	I	Bp	Ax	IDC	Luminal	×	○	○
10	74	T1bN1M0	IIA	Bp	SNB	IDC	Luminal	○	○	○

RT: radiation therapy; Bp: breast-conserving therapy; SNB: sentinel lymph node biopsy; Ax: axillary lymph node dissection; IDC: invasive ductal carcinoma; DCIS: ductal carcinoma in situ; HER2: human epidermal growth factor receptor 2; ○: administration; ×; not administered.

**Table 2:** Age at diagnosis of contralateral breast cancer, surgical method, and pathological examination.

No of Case	Years to Contralateral Breast Cancer (Years)	Age at Diagnosis of Contralateral Breast Cancer	TNM	Stage	Procedure	Axillary Lymph Node	Histology	Subtype
1	8	63	T1cN0	I	Bt	SNB	IDC	LuminalHER2
2	3	74	T1micN0	I	Bp	SNB	Apocrine	TNBC
3	10	78	T1cN0	I	Bp	SNB	ILC	Luminal
4	9	79	TisN0	0	Bt	SNB	DCIS	TNBC
5	8	76	T1micN0	I	Bt	SNB	IDC	Luminal
6	9	69	T2N0	IIA	Bp	SNB	IDC	Luminal
7	2	65	T1cN0	I	Bp	SNB	IDC	TNBC
8	6	46	T1cN0	I	Bt	SNB	IDC	Luminal
9	8	74	T1aN0	I	Bt	SNB	IDC	TNBC
10	8	82	TisN0	0	Bt	SNB	DCIS	HER2

Bt: Breast total mastectomy; Bp: breast-conserving therapy; SNB: sentinel lymph node biopsy; IDC: invasive ductal carcinoma; DCIS: ductal carcinoma in situ; TNBC: triple-negative breast cancer; HER2: human epidermal growth factor receptor 2.

**Table 3:** BI-RADS categories by image and mammary gland density in MG at the time of diagnosis of contralateral breast cancer.

No of Case	Mammographic Density	MG BI-RADS	MG Findings	US BI-RADS	MRI BI-RADS
1	Heterogeneous	2	Calcification(benign)	5	4
2	Scattered	1	No	4	4
3	Scattered	5	Mass	5	4
4	Scattered	4	Calcification	4	4
5	Heterogeneous	4	Calcification	4	4
6	Heterogeneous	5	Mass	4	4
7	Heterogeneous	4	Mass	5	4
8	Heterogeneous	1	No	4	4
9	Heterogeneous	1	No	1	4
10	Heterogeneous	1	No	1	1

BI-RADS: breast imaging reporting and data system; MG: mammography; US: ultrasonography; MRI: Magnetic Resonance Imaging.

**Table 4:** Malignant possibility and diagnosis using the AI system when diagnosing breast cancer.

No of Case	MLO, %	CC, %	AI Diagnosis
1	44.6	68.9	Malignancy
2	6.9	77.0	Malignancy
3	3.4	50.2	Malignancy
4	68.5	65.9	Malignancy
5	66.5	37.5	Malignancy
6	30.2	42.5	Malignancy
7	2.6	4.0	No
8	9.9	2.2	No
9	13.8	27.5	No
10	14.0	17.4	No

MLO: mediolateral oblique; CC: craniocaudal; AI: artificial intelligence.

**Table 5:** Analysis of MG by AI in the year before the time of diagnosis.

No of Case	Duration Since Diagnosing MG	Previous MLO, %	Previous CC, %	AI Diagnosis
1	1Y3M	88.5	77.0	Malignancy
2	1Y6M	0.1	60.9	Malignancy
3	7Y0M	5.4	1.8	No
4	1Y10M	17.6	5.5	No
5	3Y7M	19.4	19.3	No
6	1Y5M	0.6	2.3	No

MG: mammography; AI: artificial intelligence; MLO: mediolateral oblique; CC: craniocaudal.

## Data Availability

Raw data were generated at the Tokyo Medical and Dental University Hospital. The derived data supporting the findings of this study are available from the corresponding author (M.A.) on reasonable request.

## References

[1] Abdoli G., Bottai M., Sandelin K., Moradi T. (2017). Breast Cancer Diagnosis and Mortality by Tumor Stage and Migration Background in a Nationwide Cohort Study in Sweden. Breast.

[2] Bae J.M., Kim E.H. (2016). Breast Density and Risk of Breast Cancer in Asian Women: A Meta-Analysis of Observational Studies. J. Prev. Med. Public Health.

[3] Løberg M., Lousdal M.L., Bretthauer M., Kalager M. (2015). Benefits and Harms of Mammography Screening. Breast Cancer Res..

[4] Lu W., Schaapveld M., Jansen L., Bagherzadegan E., Sahinovic M.M., Baas P.C., Hanssen L.M.H.C., van der Mijle H.C.J., Brandenburg J.D., Wiggers T. (2009). The Value of Surveillance Mammography of the Contralateral Breast in Patients with a History of Breast Cancer. Eur. J. Cancer.

[5] Soerjomataram I., Louwman W.J., Lemmens V.E.P.P., De Vries E., Klokman W.J., Coebergh J.W.W. (2005). Risks of Second Primary Breast and Urogenital Cancer Following Female Breast Cancer in the South of The Netherlands, 1972–2001. Eur. J. Cancer.

[6] Chaudary M.A., Millis R.R., Hoskins E.O.L., Halder M., Bulbrook R.D., Cuzick J., Hayward J.L. (1984). Bilateral Primary Breast Cancer: A Prospective Study of Disease Incidence. Br. J. Surg..

[7] Hartman M., Czene K., Reilly M., Adolfsson J., Bergh J., Adami H.O., Dickman P.W., Hall P. (2007). Incidence and Prognosis of Synchronous and Metachronous Bilateral Breast Cancer. J. Clin. Oncol..

[8] Robertson C., Ragupathy S.K.A., Boachie C., Fraser C., Heys S.D., MacLennan G., Mowatt G., Thomas R.E., Gilbert F.J. (2011). Surveillance Mammography for Detecting Ipsilateral Breast Tumour Recurrence and Metachronous Contralateral Breast Cancer: A Systematic Review. Eur. Radiol..

[9] Smith T.J., Davidson N.E., Schapira D.V., Grunfeld E., Muss H.B., Vogel V.G., Somerfield M.R. (1999). American Society of Clinical Oncology 1998 Update of Recommended Breast Cancer Surveillance Guidelines. Am. Soc. Clin. Oncol..

[10] Kobayashi Y., Masuda K., Hiraswa A., Takehara K., Tsuda H., Watanabe Y., Oda K., Nagase S., Mandai M., Okamoto A. (2022). Current Status of Hereditary Breast and Ovarian Cancer Practice among Gynecologic Oncologists in Japan: A Nationwide Survey by the Japan Society of Gynecologic Oncology (JSGO). J. Gynecol. Oncol..

[11] Lehman C.D., Wellman R.D., Buist D.S.M., Kerlikowske K., Tosteson A.N.A., Miglioretti D.L. (2015). Diagnostic Accuracy of Digital Screening Mammography with and without Computer-Aided Detection. JAMA Intern Med..

[12] Pacilè S., Lopez J., Chone P., Bertinotti T., Grouin J.M., Fillard P. (2020). Improving Breast Cancer Detection Accuracy of Mammography with the Concurrent Use of an Artificial Intelligence Tool. Radiol. Artif. Intell..

[13] Lauritzen A.D., Rodríguez-Ruiz A., von Euler-Chelpin M.C., Lynge E., Vejborg I., Nielsen M., Karssemeijer N., Lillholm M. (2022). An Artificial Intelligence–Based Mammography Screening Protocol for Breast Cancer: Outcome and Radiologist Workload. Radiology.

[14] Dembrower K., Wåhlin E., Liu Y., Salim M., Smith K., Lindholm P., Eklund M., Strand F. (2020). Effect of Artificial Intelligence-Based Triaging of Breast Cancer Screening Mammograms on Cancer Detection and Radiologist Workload: A Retrospective Simulation Study. Lancet Digit. Health.

[15] Rodriguez-Ruiz A., Lång K., Gubern-Merida A., Teuwen J., Broeders M., Gennaro G., Clauser P., Helbich T.H., Chevalier M., Mertelmeier T. (2019). Can We Reduce the Workload of Mammographic Screening by Automatic Identification of Normal Exams with Artificial Intelligence? A Feasibility Study. Eur. Radiol..

[16] Larsen M., Aglen C.F., Lee C.I., Hoff S.R., Lund-Hanssen H., Lång K., Nygård J.F., Ursin G., Hofvind S. (2022). Artificial Intelligence Evaluation of 122969 Mammography Examinations from a Population-Based Screening Program. Radiology.

[17] Lång K., Hofvind S., Rodríguez-Ruiz A., Andersson I. (2021). Can Artificial Intelligence Reduce the Interval Cancer Rate in Mammography Screening?. Eur. Radiol..

[18] Byng D., Strauch B., Gnas L., Leibig C., Stephan O., Bunk S., Hecht G. (2022). AI-Based Prevention of Interval Cancers in a National Mammography Screening Program. Eur. J. Radiol.

[19] German Consortium for Hereditary Breast and Ovarian Cancer (2002). Comprehensive analysis of 989 patients with breast or ovarian cancer provides BRCA1 and BRCA2 mutation profiles and frequencies for the German population. Int. J. Cancer.

[20] Simonyan K., Zisserman A. (2014). Very Deep Convolutional Networks for Large-Scale Image Recognition. arXiv.

[21] Kim H.E., Cosa-Linan A., Santhanam N., Jannesari M., Maros M.E., Ganslandt T. (2022). Transfer Learning for Medical Image Classification: A Literature Review. BMC Med. Imaging.

[22] Holland K., van Gils C.H., Mann R.M., Karssemeijer N. (2017). Quantification of Masking Risk in Screening Mammography with Volumetric Breast Density Maps. Breast Cancer Res. Treat..

[23] Kanda Y. (2013). Investigation of the Freely Available Easy-to-Use Software “EZR” for Medical Statistics. Bone Marrow Transpl..

[24] Ranieri J., Guerra F., Di Giacomo D. (2020). Role of Metacognition Thinking and Psychological Traits in Breast Cancer Survivorship. Behav. Sci..

[25] Barat M., Pellat A., Hoeffel C., Dohan A., Coriat R., Fishman E.K., Nougaret S., Chu L., Soyer P. (2024). CT and MRI of Abdominal Cancers: Current Trends and Perspectives in the Era of Radiomics and Artificial Intelligence. Jpn. J. Radiol..

[26] Tatsugami F., Nakaura T., Yanagawa M., Fujita S., Kamagata K., Ito R., Kawamura M., Fushimi Y., Ueda D., Matsui Y. (2023). Recent Advances in Artificial Intelligence for Cardiac CT: Enhancing Diagnosis and Prognosis Prediction. Diagn. Interv. Imaging.

[27] Yardimci A.H., Kocak B., Sel I., Bulut H., Bektas C.T., Cin M., Dursun N., Bektas H., Mermut O., Yardimci V.H. (2023). Radiomics of Locally Advanced Rectal Cancer: Machine Learning-Based Prediction of Response to Neoadjuvant Chemoradiotherapy Using Pre-Treatment Sagittal T2-Weighted MRI. Jpn. J. Radiol..

[28] Fujima N., Kamagata K., Ueda D., Fujita S., Fushimi Y., Yanagawa M., Ito R., Tsuboyama T., Kawamura M., Nakaura T. (2023). Current State of Artificial Intelligence in Clinical Applications for Head and Neck MR Imaging. Magn. Reson. Med. Sci..

[29] Du G., Zeng Y., Chen D., Zhan W., Zhan Y. (2023). Application of Radiomics in Precision Prediction of Diagnosis and Treatment of Gastric Cancer. Jpn. J. Radiol..

[30] Hirata K., Kamagata K., Ueda D., Yanagawa M., Kawamura M., Nakaura T., Ito R., Tatsugami F., Matsui Y., Yamada A. (2023). From FDG and beyond: The Evolving Potential of Nuclear Medicine. Ann. Nucl. Med..

[31] Ozaki J., Fujioka T., Yamaga E., Hayashi A., Kujiraoka Y., Imokawa T., Takahashi K., Okawa S., Yashima Y., Mori M. (2022). Deep Learning Method with a Convolutional Neural Network for Image Classification of Normal and Metastatic Axillary Lymph Nodes on Breast Ultrasonography. Jpn. J. Radiol..

[32] Goto M., Sakai K., Toyama Y., Nakai Y., Yamada K. (2023). Use of a Deep Learning Algorithm for Non-Mass Enhancement on Breast MRI: Comparison with Radiologists’ Interpretations at Various Levels. Jpn. J. Radiol..

[33] Satake H., Ishigaki S., Ito R., Naganawa S. (2022). Radiomics in Breast MRI: Current Progress toward Clinical Application in the Era of Artificial Intelligence. Radiol. Medica.

[34] Nara M., Fujioka T., Mori M., Aruga T., Tateishi U. (2023). Prediction of Breast Cancer Risk by Automated Volumetric Breast Density Measurement. Jpn. J. Radiol..

[35] Uematsu T., Nakashima K., Harada T.L., Nasu H., Igarashi T. (2023). Comparisons between Artificial Intelligence Computer-Aided Detection Synthesized Mammograms and Digital Mammograms When Used Alone and in Combination with Tomosynthesis Images in a Virtual Screening Setting. Jpn. J. Radiol..

[36] Schaffter T., Buist D.S.M., Lee C.I., Nikulin Y., Ribli D., Guan Y., Lotter W., Jie Z., Du H., Wang S. (2020). Evaluation of Combined Artificial Intelligence and Radiologist Assessment to Interpret Screening Mammograms. JAMA Netw. Open.

